# Effect of SMS Ward Round Notifications on Inpatient Experience in Acute Medical Settings: Retrospective Cohort Study

**DOI:** 10.2196/57470

**Published:** 2025-03-12

**Authors:** Jongchan Lee, Soyeon Ahn, Jung Hun Ohn, Eun Sun Kim, Yejee Lim, Hye Won Kim, Hee-Sun Park, Jae Ho Cho, Sun-wook Kim, Jiwon Ryu, Jihye Kim, Hak Chul Jang, Nak-Hyun Kim

**Affiliations:** 1 Department of Internal Medicine, Seoul National University Bundang Hospital, Seoul National University College of Medicine, 82, Gumi-ro 173 Beon-gil, Bundang-gu, Gyeonggi-do, Seongnam-si, 13620, Republic of Korea, 82 317877085; 2 Department of Statistics, Seoul National University Bundang Hospital, Seoul National University College of Medicine, Seongnam-si, Republic of Korea; 3 Department of Hospital Medicine, Seoul St. Mary’s Hospital, Seoul, Republic of Korea

**Keywords:** rounds, round-time notification, text messaging, patient experience assessment, patient experiences, patient-centeredness, patient participation

## Abstract

**Background:**

Ward rounds are an essential component of inpatient care. Patient participation in rounds is increasingly encouraged, despite the occasional complicated circumstances, especially in acute care settings.

**Objective:**

This study aimed to evaluate the effect of real-time ward round notifications using SMS text messaging on the satisfaction of inpatients in an acute medical ward.

**Methods:**

Since January 2021, a service implementing real-time ward round notifications via text messaging (WR-SMS) has been operational at a tertiary-care medical center in Korea. To assess its impact, we conducted a retrospective cohort study of patients admitted to the acute medical unit who participated in a patient experience survey. Patient satisfaction was compared between patients admitted in 2020 (pre–WR-SMS group) and 2021 (post–WR-SMS group).

**Results:**

From January 2020 to December 2021, a total of 100 patients were enrolled (53 patients in the pre–WR-SMS group and 47 patients in the post–WR-SMS group). Compared with the pre–WR-SMS group, the post–WR-SMS group showed significantly greater satisfaction about being informed about round schedules (mean 3.43, SD 0.910 vs mean 3.89, SD 0.375; *P*<.001) and felt more emotionally supported during admission (mean 3.49, SD 0.800 vs mean 3.87, SD 0.397; *P*<.001). Regarding other questionnaire scores, the post–WR-SMS group showed an overall, although statistically insignificant, improvement compared with the pre–WR-SMS group.

**Conclusions:**

Real-time round notifications using a user-friendly SMS may improve inpatient satisfaction effectively.

## Introduction

Inpatient ward rounds constitute a salient core activity of the daily care of hospitalized patients and present a critical opportunity to deliver patient-centered care [1,2]. Ward rounds are a complex process that involves organizing the clinical care of inpatients, including diagnostic assessment and therapeutic planning by the care team [3]. For the patient, inpatient ward rounds provide an opportunity for direct patient-clinician communication, sharing of information, and participation in their own care planning [3,4]. Despite the widely recognized importance of ward rounds, an environment conducive to conducting such rounds and effectively communicating with patients is not always achievable, resulting in ineffective communication with and dissatisfaction of patients [5]. Especially in acute care settings, the participation of patients in ward rounds is likely to be limited not only due to their acute illness and fatigue [6] but also by the acute care context itself and time constraints of the ward staff [7,8].

With the emphasis on patient-centeredness, the Korean Ministry of Health and Welfare (MOHW) and the Health Insurance Review and Assessment Service (HIRA) developed the Korean Patient Experience Survey, to gain insight into the needs of inpatients and improve inpatient care quality. This survey has been conducted biennially by telephone since 2017 among patients discharged from hospitals, and the results have repeatedly revealed that patients remain least satisfied with inpatient ward rounds [9,10]. In line with global trends, studies from the United States and the United Kingdom highlight the importance of improving patient communication and providing clear, predictable information to enhance patient satisfaction [11-13]. Furthermore, evidence suggests that interventions focusing on real-time communication [14], such as the use of SMS text messaging, can help alleviate patient dissatisfaction by improving the timeliness and transparency of ward rounds.

As part of the efforts to improve patients’ satisfaction, Seoul National University Bundang Hospital (SNUBH), a 1300-bed tertiary referral hospital in the Republic of Korea, has implemented the real-time ward round notifications via text messaging (WR-SMS) service since January 2021 to inform the patients and their carers about the start of daily ward rounds, and thereby alleviating arbitrary waiting for rounds.

In 2015, a hospitalist-run acute medical unit (AMU) was established to enhance patient safety and care efficiency for patients with acute medical conditions. Although the AMU improved patient outcomes and efficiency of care [15,16], patients’ perceptions of inpatient care were found to be inconsistent, which could be attributed to the acuteness and complexity of clinical conditions, the hectic hospital environment, and heightened levels of anxiety.

As mobile devices continue to advance, SMS text messaging has been suggested to enhance clear and efficient communication in various clinical contexts [14,17]. However, there remains a gap in research regarding the potential impact of informing patients about round times via SMS text messages on communication effectiveness and patient satisfaction. This study aimed to determine the effect of real-time WR-SMS service on the satisfaction of AMU inpatients.

## Methods

### Study Design and Clinical Setting

This retrospective cohort study was conducted at the AMU of SNUBH, a hospitalist-run 46-bed ward caring for patients with acute medical conditions, who were admitted from the emergency or outpatient departments for active acute medical care, unless being hemodynamically unstable requiring invasive monitoring and critical care or terminally ill with cancer requiring only palliative care. After receiving active acute care, patients were either discharged or transferred to a specialty ward for further treatment. The AMU was operated 24 hours a day and 7 days a week by 10 board-certified medical hospitalists on a weekly rotation basis.

The SNUBH patient-experience survey has been routinely performed by telephone since 2006. Patients eligible for the survey were randomly selected within 2 to 56 days (8 wk) after discharge. Patients unable to communicate effectively due to conditions resulting in severe cognitive or communication impairments were excluded from the survey. Following the introduction of the official Korean Patient Experience Survey developed by the Korean MOHW/HIRA in 2017, the SNUBH patient-experience survey used the very same questionnaire to assess patients’ hospital experiences.

### Study Participants

Adult patients ≥19 years of age who were admitted to the AMU of SNUBH between January 2020 and December 2021 for ≥1 day and who participated in the SNUBH patient-experience survey after discharge were included. Their demographic data were retrospectively collected from the electronic medical record system, and their satisfaction was assessed based on the SNUBH patient-experience survey results. To determine whether patient satisfaction improved, we compared the scores from 2020 (before the implementation of the WR-SMS, pre–WR-SMS group) with those of 2021 (after the implementation of the WR-SMS post–WR-SMS group).

### Real-Time Ward Round Notification Service With Text Messaging

The WR-SMS service was first developed in September 2020. It was actively implemented in January 2021, after an introductory period of 3 months (October to December 2020) during which the staff was educated about and encouraged to use the service via campaigns and email or SMS notifications.

The WR-SMS service was embedded into the SNUBH electronic health record system, enabling the doctors to send short text messages (80 to 160 bytes; ie, SMS) to their inpatients directly from the electronic health record system with a few clicks. From their patient list, doctors were able to select or deselect the patients who would receive the SMS text message. The default content of the SMS text message read, “Ward rounds by Dr. OOO will begin shortly. Visiting times may vary depending on the room.” Further adjustments to the content of the message could be made as needed by the doctor. The notification could also be sent simultaneously to other relevant medical staff. The doctors were encouraged to send this WR-SMS to their patients just before starting their ward rounds, usually right after reviewing the patients’ records.

### Patient-Experience Survey

The SNUBH patient-experience survey questionnaire, which uses the same questions as the official Korean Patient Experience Survey developed by the Korean MOHW/HIRA, comprises 19 questions organized into 5 domains about the patients’ hospital experience (4 questions pertaining to “Services from nurses,” 4 pertaining to “Services from physicians,” 5 pertaining to “Medication and treatment processes,” 2 pertaining to “Hospital environment,” 4 pertaining to “Ensuring patients’ rights”, and 4 assessing patients’ demographic characteristics) (detailed questionnaire is presented in Multimedia Appendix 1). Each question was scored on a 4-point Likert scale (1=very dissatisfied, 2=dissatisfied, 3=satisfied, and 4=very satisfied) to assess patients’ subjective satisfaction with the quality of inpatient services. Except for the question about providing information about plans after discharge, answers were either “Yes” or “No.” For this study, the responses to the “Services from physicians” and “Medication and treatment processes” domain, which are closely related to inpatient ward rounds, were considered.

### Statistical Analysis

Data reflecting patients’ baseline characteristics were expressed as a median with ranges for continuous variables and as frequencies and percentages for categorical variables. Data from Likert scales were expressed as means and SDs, as well as median with ranges (IQRs). Comparisons were performed using the Mann-Whitney *U* test, Student *t* test, and *χ*² test. All tests were 2-sided and performed at a significance level of .05. Statistical analyses were performed using IBM SPSS v. 21.0 (IBM Corp).

### Ethical Considerations

The study was conducted in accordance with the Declaration of Helsinki, revised in 2013. The SNUBH institutional review board approved this study design and waived the need for obtaining informed consent from the participants due to the retrospective design (B-2203-746-101). Participant confidentiality and anonymity were strictly maintained throughout the research process, and data were handled in a secure manner to protect their privacy. In addition, the study ensured that no undue harm or burden was placed on participants and that their rights, safety, and well-being were prioritized at all times.

## Results

### Baseline Characteristics of the Participants

A total of 100 patients were enrolled from January 2020 to December 2021 (53 in the pre–WR-SMS group before WR-SMS implementation and 47 in the post–WR-SMS group after WR-SMS implementation). The median age of the patients was 59.5 (range 25‐81) years, and of the total, 59 were female. A total of 74 patients were hospitalized because of malignancy and 26 patients were admitted for non-cancer illnesses. A total of 44 and 56 patients were admitted from the emergency and outpatient departments, respectively. The median length of hospital stay was 7 (range 3‐30) days. No statistically significant difference of patients’ baseline characteristics between the pre- and post–WR-SMS groups was observed (Table 1).

**Table 1.** Baseline demographic and clinical characteristics of study participants.

Characteristics		Pre–WR-SMS group^a^ (n=53)	Post–WR-SMS group^b^ (n=47)	*P* value
Age (year), median (range)		58 (25-81)	60 (38-76)	.90
Sex, n (%)				.36
	Male	19 (36)	22 (47)	
	Female	34 (64)	25 (53)	
Disease, n (%)				.43
	Malignancy	37 (70)	37 (79)	
	Non-cancer illness	16 (30)	10 (21)	
Hospitalization route, n (%)				.94
	Emergency department	24 (45)	20 (43)	
	Outpatient department	29 (55)	27 (57)	
Education, n (%)				.25
	≤High school	29 (55)	32 (68)	
	≥College	24 (45)	15 (32)	
Length of stay (days), median (range)		7 (3-30)	7 (3-23)	.76

a Pre–WR-SMS group: patients hospitalized at the acute medical unit in 2020, before the implementation of ward round notifications via text messaging service.

b Post–WR-SMS group: patients hospitalized at the acute medical unit in 2021, after the implementation of ward round notifications via text messaging.

### Effects of Real-Time Round Notification on Satisfaction of Hospitalized Patients

**Figure 1.**
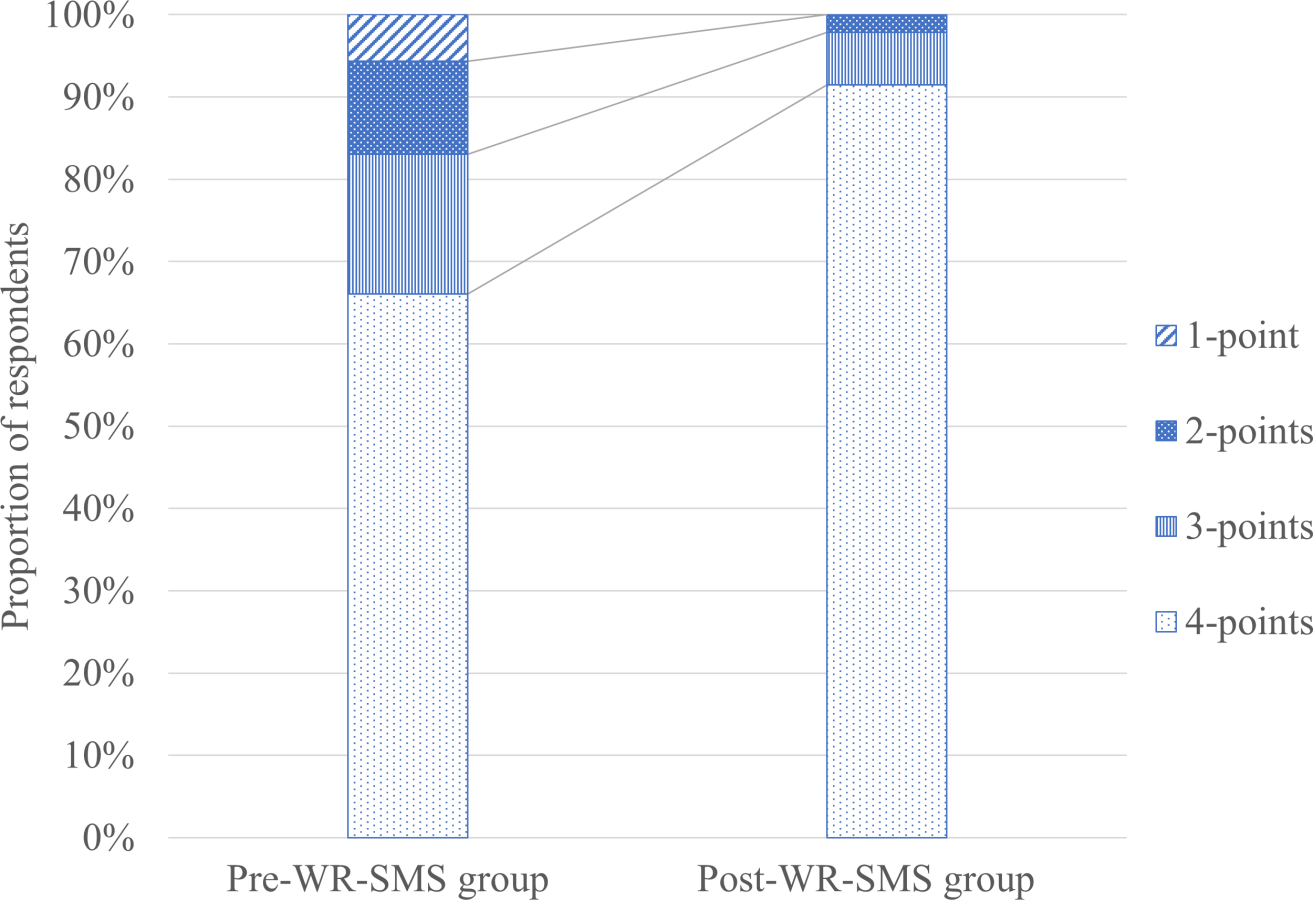
Changes in Likert scale scores regarding the provision of patient information on round schedules before and after the implementation of ward round notifications via text messaging. Pre–WR-SMS group: patients hospitalized at the acute medical unit in 2020, before the implementation of ward round notifications via text messaging service; Post–WR-SMS group: patients hospitalized at the acute medical unit in 2021, after the implementation of ward round notifications via text messaging service.

After the WR-SMS implementation in 2021, the score for the question pertaining to whether the patient had received information about round schedules improved significantly compared with 2020. Differences in the Likert scale for this question are illustrated in Figure 1. The proportion of patients who assigned a rating of 4 points (very satisfied) increased from 66% (35/53) in the pre–WR-SMS group to 92% (43/47) in the post–WR-SMS group, while the proportion of those who assigned a rating of 1 point (very dissatisfied) decreased from 6% (3/53) in the pre–WR-SMS group to 0% in in the post–WR-SMS group.

Table 2 summarizes the responses for the “Services from physicians” and “Medication and treatment processes” domains. Naturally, patients in the post–WR-SMS group showed significantly higher satisfaction about being informed about round schedules (mean 3.43, SD 0.910 vs mean 3.89, SD 0.375; *P*<.001) compared with patients in the pre–WR-SMS group. Interestingly, patients in the post–WR-SMS group also demonstrated significantly higher satisfaction with the emotional support (comfort and empathy) they received during admission (mean 3.49, SD 0.800 vs mean 3.87, SD 0.397; *P*<.001). Although not statistically significant, they were also satisfied with the description of the treatment processes (mean 3.62, SD 0.713 vs mean 3.83, SD 0.481; *P*=.09) and the explanations of possible side effects during treatment (mean 3.60, SD 0.743 vs mean 3.85, SD 0.465; *P*=.05). For all other questions in the “Services from physicians” and “Medication and treatment processes” domain, the post–WR-SMS group recorded a higher overall score compared to the pre–WR-SMS group, although it was not statistically significant (Multimedia Appendix 2).

**Table 2.** Survey results on patient satisfaction before and after the implementation of ward round notification using SMS text messaging service^a^.

Parameter			Pre–WR-SMS group^b^ (n=53)	Post–WR-SMS group^c^ (n=47)	*t* test (df)	*P* value	
Services from physicians							
	Courtesy and respect for patients				–0.93 (83.47)		
		Median (IQR)	4 (4-4)	4 (4-4)		.65	
		Mean (SD)	3.81 (0.557)	3.89 (0.312)		.36	
	Careful listening to patients				–0.47 (88.02)		
		Median (IQR)	4 (4-4)	4 (4-4)		.88	
		Mean (SD)	3.83 (0.545)	3.87 (0.337)		.64	
	Opportunities for patients to meet their doctors				–1.58 (94.08)		
		Median (IQR)	4 (3-4)	4 (4-4)		.15	
		Mean (SD)	3.58 (0.663)	3.77 (0.476)		.12	
	Receipt of information about round schedules				–3.37 (70.90)		
		Median (IQR)	4 (3-4)	4 (4-4)		.002	
		Mean (SD)	3.43 (0.910)	3.89 (0.375)		<.001	
Medication and treatment process							
	Detailed description of treatment processes				–1.72 (91.73)		
		Median (IQR)	4 (3-4)	4 (4-4)		.07	
		Mean (SD)	3.62 (0.713)	3.83 (0.481)		.09	
	Easy-to-understand explanation of potential side effects				–2.02 (88.60)		
		Median (IQR)	4 (3-4)	4 (4-4)		.045	
		Mean (SD)	3.60 (0.743)	3.85 (0.465)		.047	
	Appropriate pain relief management				–1.15 (61.49)		
		Median (IQR)	4 (4-4)	4 (4-4)		.23	
		Mean (SD)	3.86 (0.462)	3.95 (0.216)		.26	
	Comfort and sympathy regarding illness				–3.08 (78.06)		
		Median (IQR)	4 (3-4)	4 (4-4)		.003	
		Mean (SD)	3.49 (0.800)	3.87 (0.397)		<.001	
	Receipt of information on postdischarge care, n (%)		48 (90.6)	42 (89.4)	—^d^	.89	

a Values in the first row for each item represent medians with IQRs and *P* values from the Mann-Whitney test. Values in the second row for each item represent means with SDs and *P* values from the Student *t* test (2-tailed).

b Pre–WR-SMS group: patients hospitalized at the acute medical unit in 2020, before the implementation of ward round notifications via text messaging service.

c Post–WR-SMS group: patients hospitalized at the acute medical unit in 2021, after the implementation of ward round notifications via text messaging service.

d Not applicable.

## Discussion

### Principal Findings

The WR-SMS implementation resulted in significant improvement of inpatient satisfaction among patients hospitalized in the AMU. Besides the direct improvement in scores pertaining to the receipt of round schedule information (mean 3.43, SD 0.910 vs mean 3.89, SD 0.375; *P*<.001), patients felt more emotionally supported and involved in the care process (mean 3.49, SD 0.800 vs mean 3.87, SD 0.397; *P*<.001), and also expressed higher satisfaction with the explanations provided about treatment side effects (mean 3.60, SD 0.743 vs mean 3.85, SD 0.465; *P*=.047). These significant findings emphasize the value of real-time communication on both procedural and emotional aspects of care. Our results indicate that by enabling patients to participate more actively in routine ward rounds, WR-SMS fosters a greater sense of involvement and satisfaction, reaffirming the critical role of patient-centered communication in inpatient care.

Patient-centeredness is an essential component of high-quality health care, contributing to improved experience and clinical outcomes [18]. It is defined by the Institute of Medicine as the establishment of a partnership between the patient and health care providers to ensure that the wishes, needs, and preferences of the former are respected in the shared decision-making process [19]. Since the early 2000s, many countries have implemented patient-reported experience measures (such as standardized surveys) at the national level. The data are publicly reported, in order to assess and monitor patients’ health care experiences and improve the quality of care [11,12]. For example, the United States Hospital Consumer Assessment of Healthcare Providers and Systems (HCAHPS) survey, first implemented in 2006, measures patients’ perspectives on the quality of care they receive during their hospital stay, with its scores influencing hospital payment [13]. The United Kingdom and the Netherlands also conduct national-level surveys to assess patient satisfaction, which are used for various purposes related to improving health care services [20,21]. Previous studies revealed that quality improvement activities were initiated after patient care performance data were publicly released [22], with even hospitals that exhibited high levels of patient satisfaction delivering higher-quality clinical care [13].

In South Korea, the Korean Patient Experience Survey has been regularly conducted by HIRA biennially since 2017 by telephone among patients discharged from hospitals. It was developed based on the American HCAHPS, but further modified to suit the Korean context and to promote improvement of current Korean health care service quality [23]. A 2015 survey by the Korea Institute for Health and Social Affairs revealed that 30.5% of hospitalized patients claimed not to have been adequately informed about ward rounds and 20.4% reported not being included in the care process [24]. Furthermore, qualitative research during the Korean Patient Experience Survey development process revealed that inpatients were frequently unsatisfied with the communication with their doctors, especially regarding the lack of advance notice and unpredictability of ward round times [23]. To gauge these concerns, a direct question about whether the patient has been informed about the ward round time was included in the official Korean Patient Experience Survey.

Despite the increasing emphasis on patient-centeredness, patients are often still excluded from their own care process [5,6]. The results of the previous Korean Patient Experience Surveys (first to third, conducted from 2017 to 2021) reveal that the scores for the “Ensuring patients’ rights” and “Services from physicians” domains remain lowest [9,10,25], suggesting that communication between patients and doctors remains unsatisfactory and patients do not feel sufficiently involved in the care process. Among several factors influencing patient participation and communication, such as the ward staff’s attitude, time constraints [7], and health literacy [26], the care environment context itself has been identified as critical [8]. Previous Korean patient surveys revealed that patients felt dissatisfied and even frustrated by the medical staff’s impolite, indifferent, or authoritarian attitudes and faced difficulties in communicating with their doctors who always appeared busy, and took issue with unpredictable and irregular round times [23]. Particularly in acute care environments, the patients’ ability to participate is further diminished by their vulnerability, illness, and anxiety; furthermore, even the ward atmosphere is often disorderly and far from suitable for effective communication. In such situations, patient involvement is notably infrequent [6].

Patients in our AMU were mostly afflicted with complex, acute illness and frequently in need of interventional procedures, imaging studies, and even surgical procedures. Therefore, these patients and their carers often missed routine ward rounds during planned procedures or did not notice that ward rounds were taking place. Furthermore, the acuteness of the patients’ conditions and lack of awareness about ward rounds likely led to frustration, anxiety, and dissatisfaction of patients and their carers toward their care. Real-time notifications provided through the WR-SMS addressed these issues by increasing awareness and predictability of ward rounds, which may help mitigate the emotional burden associated with the uncertainty of acute care. This aligns with studies suggesting that structured and predictable communication mechanisms, such as SMS notifications, create a more conducive environment for patient participation in care processes. A study conducted by Redley et al [6] at an acute inpatient ward indicated that only 18% (20/52) of rounds involved patient participation in clinical decisions, although even the patients who participated in rounds indicated low preferences for participation upon arrival at the hospital. The authors suggest that the patients’ preferences for participation could change during the course of their hospital stay and it is also likely that passive participation may have been driven by limited opportunities to participate [6]. Similarly, Walton et al [27] revealed that familiarity with the hospital environment tended to positively affect participation in ward rounds, with several participants highlighting the uncertainty regarding the timing of rounds as a source of difficulty and anxiety.

With the development of communication devices such as cellular phones and smartphones, text messaging via SMS is increasingly used to enhance effective communication with patients and carers [14,17]. In our institution, the WR-SMS was developed and introduced to inform inpatients about the start of ward rounds in real time to improve their opportunities to participate in them. As physicians typically review their patients’ medical records right before they begin ward rounds, the WR-SMS was embedded into our electronic health record system to enhance its use instead of using a separate platform. Following its implementation in January 2021, its utilization rate rapidly increased with long-term promotional activities, reaching an average of 65% in 2021. With its use, although only a statistically insignificant increase in patients’ perceptions of opportunities to meet their doctors was noted (possibly owing to the small sample size and the nature of the ward, which is staffed by hospitalists who are primarily based within the unit), inpatient satisfaction significantly improved with regard to “providing information about round-time” and the “Medication and treatment processes” domain. However, scores for other aspects in the “Services from physicians” domain did not improve significantly, suggesting that while real-time SMS notifications enhance patients’ involvement and satisfaction with specific aspects of care, they may need to be supplemented with other interventions to address broader communication gaps.

### Limitations

To our knowledge, this is the first study to investigate the effect of real-time round notification via SMS on inpatients’ satisfaction. Nevertheless, it has some limitations. First, it was conducted at a single referral center in South Korea. As of 2019, the penetration rate of mobile phones in South Korea was 100%, with smartphones accounting for 95%. Therefore, our results may not be reproduced in other countries with lower mobile phone penetration rates. Second, as we used information from subjects who participated in the SNUBH patient-experience survey, instead of conducting a new survey, the number of patients included in this study is relatively small, precluding further adjustments and analyses based on clinical characteristics or comorbidities. However, the clinical setting remained constant throughout the study period and the subjects for the SNUBH patient-experience survey were randomly selected, which should minimize bias. Third, since this study included only patients capable of communication as survey respondents, it was not possible to assess the impact of WR-SMS on caregiver satisfaction.

### Conclusion

Effective communication between physicians and patients, particularly during inpatient ward rounds, is a crucial aspect of inpatient care. This study proposes that real-time round notifications delivered through a user-friendly SMS service could improve inpatient satisfaction, likely by providing predictability and fostering an improved perception of emotional support. In the contemporary landscape, with personal communication devices becoming increasingly ubiquitous, the strategic utilization of such devices is anticipated to significantly influence patient satisfaction.

## Supplementary material

10.2196/57470 Multimedia Appendix 1 Questionnaire that was used for the Seoul National University Bundang Hospital patient-experience survey and the official Korean patient-experience survey developed by the Korean Ministry of Health and Welfare/Health Insurance Review and Assessment Service.

10.2196/57470 Multimedia Appendix 2 Changes in Likert scale survey scores on patient satisfaction before and after the implementation of ward round notifications via text messaging. Pre–WR-SMS group: patients hospitalized at the acute medical unit in 2020, before the implementation of ward round notifications via text messaging service; Post–WR-SMS group: patients hospitalized at the acute medical unit in 2021, after the implementation of ward round notifications via text messaging service.

## Abbreviations

AMU: acute medical unit
HCAHPS: Hospital Consumer Assessment of Healthcare Providers and Systems
HIRA: Health Insurance Review and Assessment Service
MOHW: Ministry of Health and Welfare
SNUBH: Seoul National University Bundang Hospital
WR-SMS: ward round notifications via text messaging
