# Supplementary material for: Effect of SMS Ward Round Notifications on Inpatient Experience in Acute Medical Settings: Retrospective Cohort Study
Source: JMIR Hum Factors. 2025 Mar 12;12:e57470. doi: 10.2196/57470 (PMC11922492; doi:10.2196/57470)
Supplement: Multimedia Appendix 1 [file humanfactors-v12-e57470-s001.docx]

**Table S1**. The questions addressing patients’ hospital experiences in the patient experience survey. (The same questionnaire was used for the SNUBH patient-experience survey and the official Korean patient-experience survey developed by the K-MOHW/HIRA).

| Questions | Scale |
| --- | --- |
| Services from nurses |  |
| Did the nurse in charge treat you with courtesy and respect? | 1 – 4 |
| Did the nurse in charge listen to you carefully? | 1 – 4 |
| Did the nurse in charge explain the hospital stay in an easy-to-understand manner? | 1 – 4 |
| Did the nurse in charge try to address your needs when you required assistance? | 1 – 4 |
|  |  |
| Services from physicians |  |
| Did the physician in charge treat you with courtesy and respect? | 1 – 4 |
| Did the physician in charge listen to you carefully? | 1 – 4 |
| Did you or your caregivers have frequent opportunities to meet with the attending physician and have discussions? | 1 – 4 |
| Did you receive information about the attending physician’s rounds schedule or any changes to it? | 1 – 4 |
|  |  |
| Medication and treatment processes |  |
| Did the medical staff explain the reasons for medications, tests, or procedures in an easy-to-understand manner before administering them? | 1 – 4 |
| Did the medical staff explain the potential adverse effects of medications, tests, or procedures in an easy-to-understand manner after administering them? | 1 – 4 |
| Did the medical staff employ appropriate management measures to relieve your pain? | 1 – 4 |
| Did you receive comfort and empathy regarding your condition? | 1 – 4 |
| Did you receive information about post-discharge instructions and treatment plans? | Yes/No |
|  |  |
| Hospital environment |  |
| Was the hospital environment clean and well maintained? | 1 – 4 |
| Was the hospital environment safe? | 1 – 4 |
|  |  |
| Ensuring patients’ rights |  |
| Did you receive fair treatment compared to other patients during your hospitalization? | 1 – 4 |
| Was it easy to express your dissatisfaction, if any, during your hospitalization? | 1 – 4 |
| Were you given opportunities to participate in the decision-making process for tests or treatment? | 1 – 4 |
| Were you treated with consideration to minimize any feelings of exposure or discomfort during tests or treatments? | 1 – 4 |
