# Supplementary figures and images for: Effect of SMS Ward Round Notifications on Inpatient Experience in Acute Medical Settings: Retrospective Cohort Study
Source: JMIR Hum Factors. 2025 Mar 12;12:e57470. doi: 10.2196/57470 (PMC11922492; doi:10.2196/57470)

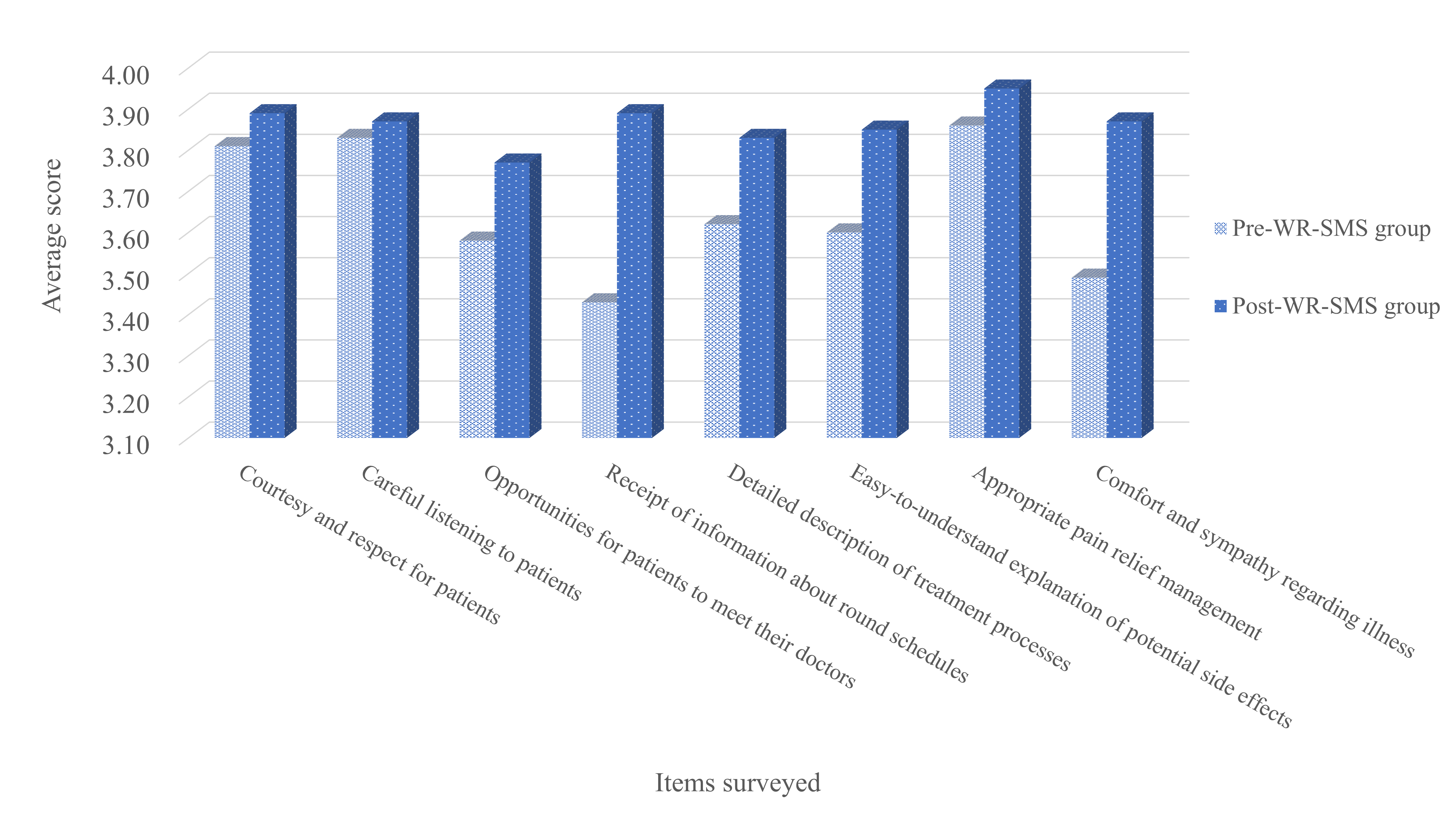

Supplement: Multimedia Appendix 2 [file humanfactors-v12-e57470-s002.png]
